# Fatal case of sorafenib-associated idiosyncratic hepatotoxicity in the adjuvant treatment of a patient with renal cell carcinoma

**DOI:** 10.1186/1471-2407-12-590

**Published:** 2012-12-11

**Authors:** BP Fairfax, S Pratap, ISD Roberts, J Collier, R Kaplan, AM Meade, AW Ritchie, T Eisen, VM Macaulay, A Protheroe

**Affiliations:** 1 Department of Oncology, Cancer and Haematology Centre, Churchill Hospital, Oxford, OX3 7LJ, UK; 2 MRC Clinical Trials Unit, London, NW1 2DA, UK; 3 Cambridge Biomedical Research Centre, Cambrige, CB2 0QQ, UK; 4 Department of Cellular Pathology, John Radcliffe Hospital, Headington, OX3 9DU, UK; 5 Department of Gastroenterology, John Radcliffe Hospital, Headington, Oxford, OX3 9DU, UK

**Keywords:** Sorafenib, Hepatotoxicity, Adjuvant, SORCE, RCC, DILI

## Abstract

**Background:**

Sorafenib is an orally available kinase inhibitor with activity at Raf, PDGFβ and VEGF receptors that is licensed for the treatment of advanced renal cell carcinoma (RCC) and hepatocellular carcinoma (HCC). Current evidence-based post-nephrectomy management of individuals with localized RCC consists of surveillance-based follow up. The SORCE trial is designed to investigate whether treatment with adjuvant sorafenib can reduce recurrence rates in this cohort.

**Case presentation:**

Here we report an idiosyncratic reaction to sorafenib resulting in fatal hepatotoxicity and associated renal failure in a 62 year-old man treated with sorafenib within the SORCE trial.

**Conclusion:**

This is the first reported case of sorafenib exposure associated fatal toxicity in the adjuvant setting and highlights the unpredictable adverse effects of novel adjuvant therapies.

## Background

The current treatment of kidney cancer that has not spread involves, when surgically feasible, the removal of the affected kidney. When the tumour is very advanced or particularly aggressive there is a high risk that despite this procedure the cancer will recur either locally or in another organ. For these individuals the current management is limited to observation of the patient and treatment if recurrence is observed. The SORCE trial aims to investigate whether giving these high-risk patients sorafenib, a drug with anti-cancer activity in kidney cancer that is known to have spread, will reduce the recurrence rate. Here we report the case of a patient on the SORCE trial who died from liver failure associated with sorafenib treatment. Although this is an extremely uncommon occurrence, this case has important implications in the treatment of patients who are asymptomatic and may indeed be cancer free as well as alerting clinicians to this rare adverse drug reaction.

## Case presentation

We describe the case of a previously fit 62 year-old man who was diagnosed with RCC after presenting with new-onset haematuria. His past medical history consisted of isolated hypertension for which he took felodopine (5mg OD) and bisoprolol (10mg OD). He was a former smoker but had no other risk factors for, or a family history of, renal cancer. He had an open nephrectomy at which there was no evidence of local or regional metastases. The excised right kidney contained an 11cm diameter clear cell RCC for which the Leibovitch score was 9 – a score associated with a 5 year metastasis free survival of 12.7% [1].

Sorafenib prolongs progression-free survival in patients with advanced RCC who have failed other treatments [2] and shows efficacy as a first-line agent [3]. Identification of treatments that reduce relapse rate or extend disease-free remission is of utmost clinical importance. The SORCE study is a phase III randomised placebo controlled double-blind study investigating the role of adjuvant sorafenib in patients with resected primary renal cell carcinoma at intermediate or high risk of relapse (Clinical trial identifier: NCT00492258). The primary outcome measure of this three-armed study is disease free survival; comparing those treated with sorafenib for either 1 year or 3 years with placebo. Secondary outcome measures include metastasis-free survival, disease-specific survival time and overall survival.

**Figure 1:**
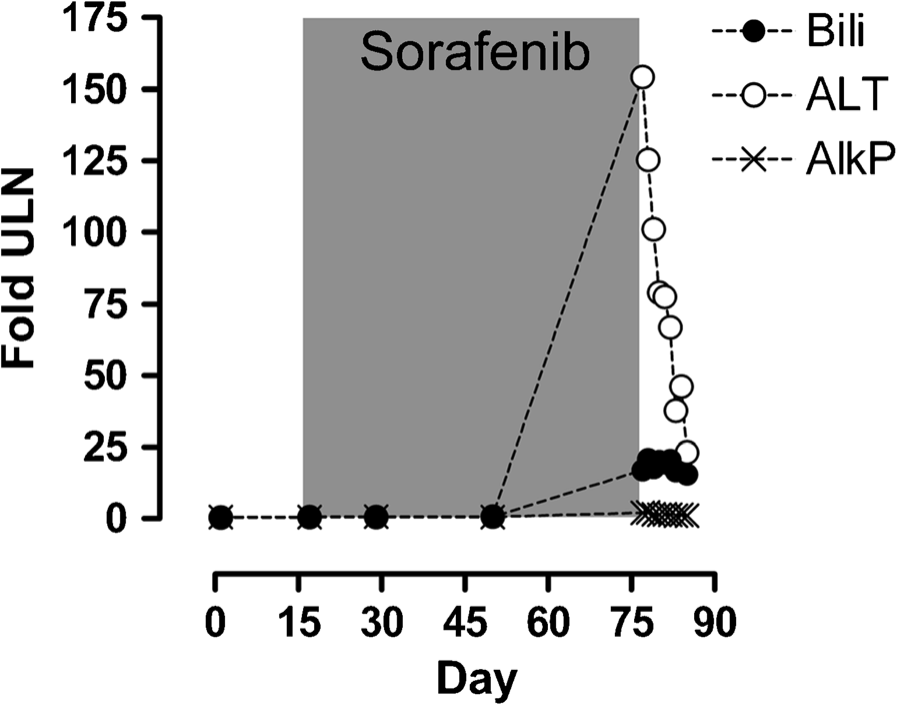
**Liver function tests on a temporal basis with the period of sorafenib treatment shaded grey expressed as fold increase on upper limit of normality.** Bili: bilirubin, ALT: alanine aminotransferose, AlkP: alkaline phosphatase, ULN: upper limit normal.

Three months post- nephrectomy the patient was enrolled into the SORCE study. At this point he was well and physically active and his blood parameters were within normal range (Figure 1). He was commenced on study medication which was later confirmed to be sorafenib 400mg BD. The initial 3 weeks of treatment were free of adverse effects, but at week 4 he noted increasing fatigue. By week 6 of treatment he had developed grade 1 mouth ulceration and grade 2 plantar erythrodysaesthesia. Additionally he suffered malaise, with gastrointestinal discomfort and mild diarrhoea and had noted an abdominal rash. He remained normotensive with a normal full blood count, normal renal function and normal liver function. By week 7 of treatment his symptoms worsened; with increasing fatigue, loss of appetite, nausea, vomiting and diarrhoea. Treatment was withdrawn but at this point jaundice developed. He had not noted change in urine colour or volume, although he complained of abdominal bloating and loose stools. There was no history of fever but he was anorexic with 2kg weight loss. Apart from his prescription anti-hypertensives and the sorafenib, he had taken no other medications including antibiotics, statins or over-the-counter analgesics. He had consumed no alcohol over the study period and his previous alcohol consumption was negligible. On examination, he was normotensive, euvolaemic and was clearly jaundiced with non-tender hepatomegaly. He was admitted to hospital at this point for further management.

### Investigations and management

Admission blood tests revealed an acute hepatitis with an ALT 6935 (normal 10-45IU), bilirubin 288μM (3-17μM), alkaline phosphatase 577IU (35-320IU) (Figure 1) and a prothrombin time of 18.2s (n=12-15s). LDH was markedly raised at 1226IU (n<260IU). The full blood count was normal without leukocytosis whilst CRP was moderately raised (34mg/dl, n<8mg/dl), a level that persisted throughout treatment. His creatinine was moderately raised at 201 μM, the baseline for this patient being 120-140 μM post-nephrectomy. An abdominal ultrasound demonstrated normal hepatic echogenicity with normal calibre common bile duct and patent hepatic vasculature. There was no obstructive uropathy. Viral screening excluded acute cytomegalovirus and Epstein Barr virus and hepatitis viruses. An immunology screen was negative for ANA and ANCA but identified complement consumption with reduced C3 (29mg/dl, 65-190mg/dl) and C4 levels (13.6mg/dl, 14-40mg/dl). A pyrexia of 37.9C was recorded on day 2 of admission and the patient was given empirical ceftriaxone, although a septic source was not identified and blood cultures were uniformly negative. There followed marked deterioration in hepatic and renal function with rising prothrombin time refractory to Vitamin K and requiring repeated plasma transfusions. On day 4, the patient became encephalopathic and was transferred to the intensive care unit where he was intubated. A repeat ultrasound revealed mild peri-hepatic ascites which was subsequently found to be transudative in nature. Further deterioration of renal function precipitated initiation of continuous veno-venous haemofiltration. Over the subsequent three days, despite maximal supportive therapy including N-acetyl-cysteine, there was progressive deterioration. His previous renal tumour excluded him from candidacy for liver transplant. Hypoglycaemia and lactataemia ensued and by day 7 the prothrombin time had risen to >200s and serum ammonium concentration was 120μmol/L (<35μmol), signifying end-stage hepatic failure, and treatment was withdrawn.

**Figure 2:**
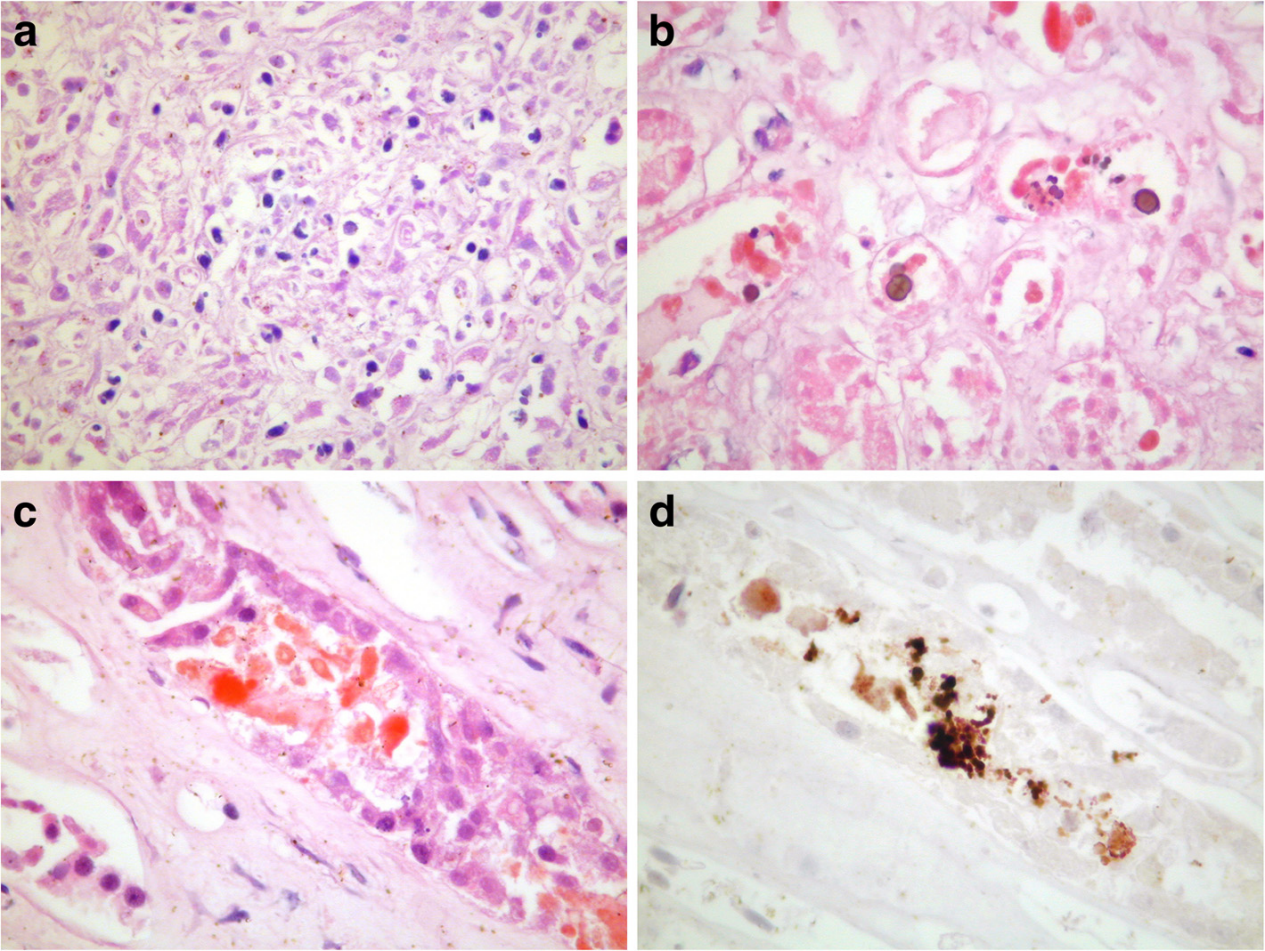
**Histology of post-mortem liver showed a lobular hepatitis with hepatocyte necrosis (a, H&E stain).** Histology of the kidneys demonstrated acute tubular necrosis with cellular debris and oxalate crystals within tubules (**b**, H&E stain) and collecting ducts (**c**, H&E stain). In addition, collecting ducts contained small numbers of myoglobin casts (**d**, immunoperoxidase stain).

At autopsy there was no evidence of metastatic disease. A terminal bronchopneumonia was noted although organisms were not isolated. Histology of post-mortem liver showed a lobular hepatitis with mononuclear cell infiltrate and hepatocyte necrosis (Figure 2a, H&E stain). Histology of the kidneys demonstrated acute tubular necrosis with cellular debris and oxalate crystals within tubules (2b, H&E stain) and collecting ducts (2c, H&E stain). In addition, collecting ducts contained small numbers of myoglobin casts (2d, immunoperoxidase stain).

## Conclusion

We believe the most likely cause of death to be an idiosyncratic allergic reaction to sorafenib manifesting as hepatotoxicity with associated renal impairment. This is supported by a RUCAM causality score of 7, consistent with sorafenib being the probable cause [4]. Drug induced liver injury (DILI) is attributable to either direct hepatotoxicity occurring soon after drug exposure, or a delayed idiosyncratic reaction. Idiosyncratic drug reactions are thought secondary to immune response, possibly to drug-protein haptens. They typically take 2–8 weeks to manifest and may demonstrate ongoing progression despite withdrawal of the offending drug [5,6].

Although sorafenib-induced hepatic dysfunction has been previously described, this is the first description of sorafenib-induced hepato-renal failure and represents the first fatality in an individual otherwise systemically well without underlying liver disease or known metastases. Sorafenib associated liver failure was first described in a phase II trial for advanced thyroid cancer [7] where the patient developed elevated LFTs 8 weeks after starting sorafenib. Despite sorafenib withdrawal, hepatic function worsened, culminating in death secondary to hepatic failure. Sorafenib hepatotoxicity was likewise reported in a 65 year old female with underlying compensated cirrhosis, secondary to non-alcoholic steatohepatitis, who received sorafenib as a third-line therapy for advanced follicular thyroid carcinoma [8]. Treatment was stopped after approximately 3 weeks when she developed a skin rash with associated fever and flu-like symptoms. At this point there was mild derangement of hepatic function which progressed rapidly over the subsequent 3 weeks, resulting in hospitalisation with a hepatocellular picture of markedly raised trans-aminases. A liver biopsy demonstrated drug-induced necrotic hepatitis and the patient responded to treatment with corticosteroids. Two further cases of sorafenib related hepatotoxicity have also been described; in the treatment of HCC [9], and also in an individual who had undergone cadaveric liver transplant for HCC [10]. However, these cases differ in that LFTs became abnormal soon after sorafenib administration (less than 5 days) and hepatic function normalised upon withdrawal of therapy. The second of these cases had a liver biopsy demonstrating hyperallergic features with hepatic inflammatory infiltrate consistent with an immune mediated process [10].

Genome wide association studies of DILI consistently demonstrate susceptibility conferred by major histo-compatibility complex (MHC) polymorphisms, supporting an immune basis to idiosyncratic DILI [11]. Although antibiotics are the most studied DILI culprits showing consistent HLA associations [12-14] similar HLA associated hepatotoxicity has been noted to small molecule kinase inhibitors. MHC associations have been reported with susceptibility to lapatanib and pazopanib hepatotoxicity [15,16]. Polymorphisms within HFE, the haemachromatosis gene residing within the extended MHC, are associated with pazopanib toxicity; whilst lapatinib toxicity is reproducibly associated with carriage of the class II HLA allele HLA_DQA1*0201.

The case we describe showed a pronounced immunoresponse with elevated CRP, low-grade pyrexia, complement consumption and an immune infiltrate in the liver. We suggest that this, coupled to a typical delay in onset between commencing sorafenib and hepatic failure, would support an immune mediated process. Given the precedents shown with other DILI, a link to HLA subtype may be anticipated. Patterns of DILI tend to be drug specific, with three phenotypes in terms of hepatic dysfunction recognised. These being a hepatocellular pattern, a cholestatic pattern and a mixed picture with derangement in both trans-aminases and cholestasis but neither picture predominating [6,17]. The pattern of liver panel abnormalities in this case and the other 2 reported cases of delayed DILI to sorafenib would suggest sorafenib can cause DILI with a hepatocellular pattern of LFTs, with an ALT>3 x upper limit of normal (ULN) and ratio of ALT/ULN: AlkP/ULN >5 between 3–7 weeks after initial exposure.

As with many idiosyncratic DILI, sorafenib hepatotoxicity is uncommon. In phase III clinical trials of sorafenib monotherapy the incidence of hepatic failure was similar amongst sorafenib and placebo recipients [18]. Similarly, in two prospective phase III clinical trials of sorafenib in patients with advanced HCC and mild liver impairment (Child-Pugh A), the incidence of hepatic failure and hepatic encephalopathy events were comparable between patients taking placebo (2.4% and 2.1%) and sorafenib (2.5% and 1.8%) [19]. Likewise hepatic failure/encephalopathy events were not observed in a placebo controlled prospective phase III study in advanced RCC (TARGET, n=903) [2]. Analysis of reported toxicities from the SORCE trial supports the rarity of sorafenib hepatotoxicity in the adjuvant setting, with this case representing an idiosyncratic reaction as opposed to a marked example of generalized hepatotoxicity (Meade A., personal communication). Nonetheless, given that a proportion of patients receiving sorafenib may have impaired liver function due to HCC or metastatic disease, it is possible a delayed drug reaction to sorafenib may be mistaken for, or coincide with disease progression. With this in mind it is noteworthy that, including this case, all episodes of sorafenib-associated acute liver failure have been reported outside HCC treatment.

This case is the first documented death directly attributable to sorafenib in an otherwise well individual. Since the patient had no evidence of metastatic disease prior to commencement of treatment or at autopsy, the case raises questions regarding the stratification of adjuvant treatments for which no current evidence exists in an outwardly healthy group. While currently available data suggests such reactions are likely to be extremely rare, we would urge clinicians to be vigilant as to possible sorafenib DILI and ensure suspected cases are reported. We suggest that future trial-wide genetic profiling of patients may potentially expedite identification of markers of idiosyncratic drug reaction to novel oncological agents, including those denoting HLA allele status [20], permitting genotype based risk stratification.

### Ethics statement

The SORCE trial is a Medical Research Council sponsored randomised double-blind study with both main UK wide Research Ethics Committee (REC) approval and local ethical approval. It is a National Cancer Institute verified trial and registered with the U.S. National Institutes of Health database ClinicalTrials.gov (Identifier: NCT00492258).

### Consent

Written informed consent was obtained from this patient’s next of kin for publication of this Case report and accompanying images. A copy of the written consent is available for review by the Series Editor of this journal.

## Competing interests

TE declares receiving research support and honoraria from Bayer as well as sitting on advisory boards and speakers bureau for Bayer. All other authors declare they have no competing interests.

## Authors’ contributions

BPF drafted the manuscript and was a member of the clinical team. SP, VM, JC & AP oversaw clinical management. ISDR provided expert advice, analysis and images. RK, AMM, AP, AWR, TE directed the SORCE trial and provided analysis of adverse effects across the cohort. AP conceived and supervised the report. All authors provided substantive intellectual contributions to and critical appraisal of the report and approved of the final manuscript.

## Pre-publication history

The pre-publication history for this paper can be accessed here:

http://www.biomedcentral.com/1471-2407/12/590/prepub
